# Aggregated imports and expenditure components in Bangladesh: A cointegration and equilibrium correction analysis

**DOI:** 10.1016/j.heliyon.2023.e17417

**Published:** 2023-06-20

**Authors:** Md Zobraj Hosen

**Affiliations:** Police Headquarters, Bangladesh Police, Dhaka, Bangladesh

**Keywords:** Import demand, Expenditure components, Global financial crisis, Cointegration, Bangladesh

## Abstract

Even though there have been a few studies on Bangladesh's aggregate import demand, the effects of the global financial crisis (GFC) on aggregate import demand still need to be measured. The short-run determinants of import demand also remained to be identified in the country. This paper explores both short-run dynamic and long-run cointegrating relationships, capturing the impact of the GFC on aggregate import demand. This study uses annual data from 1980 to 2021 (N = 42) and employs different econometric techniques for efficient results essential for compelling policy implications. The study derives an efficient dynamic equation using the best error correction mechanism. Additionally, this study includes unconventional determinants, namely, foreign currency reserves and components of expenditure (i.e., exports, private consumption and government expenditures, and expenditures on investment goods), along with the traditional import demand function. The study finds that all conventional and unconventional determinants of import demand are significant in both the long and short run. All determinants except relative price positively influence the volume of import demand. The income elasticity reduces over time, and the price inelasticity remains non-zero and negative, which indicates the competitiveness of domestic product substitutes for importable goods in the economy. In the long run, trade liberalization and foreign currency reserves have a limited positive influence on import demand. The findings of this study would be helpful for import-related policy implications in the country.

## 1 Introduction

International trade significantly impacts economic development [1,2]. Import demand is a vital part of international trade, and elasticity is essential in analyzing the state of any economy in terms of the balance of payments (BoP), exchange rate, inflation, interest rate, and gross domestic product (GDP) [1,3]. Relatively low-priced imported goods help keep the market competitive [4]. Domestic firms also tend to supply the lowest-cost production by producing at their maximum capacity to survive in the competitive market [5,6]. Therefore, people can consume low-priced goods from the market and get optimum satisfaction [4]. This competitive nature among domestic firms enables them to export more goods and earn foreign currency [7]. Imports are also a significant government revenue source through tariff and non-tariff measures [7]. Conversely, more imports may cause an unfavorable current account balance and prevent substitutable products from domestic firms [3,8].

Most studies show that imports, especially imports of intermediate goods, speed up exports and help domestic businesses [9]. For making policy decisions about imports, it is essential to know what affects import demand, such as a country's GDP, relative prices, foreign reserves, and remittance earnings. The estimated results of the factors will help policymakers make import-related policies that will help the economy grow correctly. Estimating the elasticities of total income (GDP) and the relative price or real exchange rate of import demand is not only a good idea, but the elasticity of disaggregated income (private consumption, including government spending, investment, and export) is also necessary for correct policy implications [[10], [11], [12]].

The global financial crisis (GFC) and the COVID-19 pandemic affected the growth of the world economy by causing economic instability and slowing down domestic production and international trade (exports and imports) [[13], [14], [15], [16], [17], [18], [19], [20]]. If a crisis or pandemic is the cause of this global macroeconomic instability, it might last for ten years or longer [21,22]. As a result, several countries worldwide may change their monetary and fiscal policies to get their economies back on track after the crisis [15]. This could change the elasticity of the factors that affect import demand [12,23]. A small open economy like Bangladesh, which depends a lot on imported goods (its imports have always been higher than its exports over time) [24,25], is more likely to be hurt by a global crisis and might see a change in how the import demand is affected by its factors. Thus, this study revisits the elasticities of aggregate import demand's determinants, including unconventional disaggregated income components, to identify possible changes due to the GFC so that the relevant policymakers could use those for efficient import-related policy implications in the country. Moreover, this study employs different econometric techniques, such as the Vector Autoregression (VAR) model, Engle-Granger two-step (EG) procedure, and Autoregressive Distributive Lag (ARDL) model, for efficient short- and long-run results that are essential for relevant policy implications.

## 2 Literature review

Researchers explored studies regarding the relationship between aggregated import demand and its traditional determinants (i.e., aggregate income and the relative price or exchange rate) in developed and developing countries [[26], [27], [28], [29], [30], [31], [32], [33], [34]]. However, the relationship between total imports and total income might raise the problem of aggregation bias [9,10,12,35]. Because the import demand for the components of income (i.e., total private consumption, total government expenditure, total investment, and export) in a country might differ, this is not correctly attributed to the aggregated income [9,10,12,35]. Therefore, some studies demonstrated the determinants of aggregate import demand by unconventionally disaggregating income components [3,9,10,12,35], including foreign currency reserves [3,9,35] and population growth [10], to minimize aggregation bias in the estimations. For instance, studies used national cash flow (GDP - I - G - X, where I = investment, G = government expenditure, and X = export) [36,37], the current activity variable (GDP - X) [38], and final consumption expenditure (FCG = C + G, where C = private consumption and G = government expenditures), including final expenditure on investment goods and export demand [3,39,40], as unconventionally disaggregated income determinants of import demand. In addition, some studies used foreign currency reserves as an essential determinant of import demand for a developing country because the amount of foreign currency reserves indicates the country's strength in making safe payments to foreign exporters [3,9,35].

One recent study in India showed a different elasticity between aggregated and disaggregated income components as determinants of import demand [10]. Population growth and trade openness positively impacted imports, while the exchange rate had a negative impact [10]. Another study in Türkiye found decreased elasticities of aggregated and disaggregated income components in aggregated import demand but total intermediate imported goods [12]. China's domestic income and real exchange rate elasticities of import demand were positive and negative, respectively [41]. But another study did not find a significant difference between aggregated and disaggregated income elasticities of import demand [42]. However, the elasticities differed based on various imported commodities in China [43]. A study in 32 countries in Sub-Saharan Africa (SSA) estimated that the countries' relative prices, expenditure components, and foreign currency reserves are crucial determinants of import demand [35]. Another study in five Asian countries, such as India, Japan, Korea, Singapore, and Thailand, found a significant positive influence of foreign currency reserves on import demand [44]. A recent study analyzed panel data from 19 developing countries and found an insignificant relationship between export composition, diversification, and economic growth [1]. In contrast, the study showed a significant impact of high-tech and capital goods imports on economic growth [1].

In Bangladesh, available studies demonstrated the elasticities of traditional determinants of import demand where income was positively elastic and relative import price was negatively inelastic [30,33,45]. Few studies included foreign currency reserves as a crucial unconventional determinant of import demand and found mixed results, such as positive [46] and negative [3,47,48] impacts on import demand in the country. Almost all studies found (positive) more than unit income elasticity and (negative) relative price inelasticity for import demand. But one study found positive unit elasticity of income [3], and another found a positive inelastic income elasticity of import demand based on a sample of annual data from 1978 to 2008 [30]. One study in Bangladesh investigated the long- and short-run elasticities of unconventionally disaggregated income components of import demand (i.e., foreign exchange reserves, exports, final private consumption and government expenditures, and final expenditure on investment goods) using the annual data from 1978 to 1980 [3]. The study with components of income found a positive inelastic elasticity of export from import demand, while other components of income are statistically insignificant in the long run. Moreover, the study found that relative import prices and exports are statistically significant determinants of import demand in the short run [3].

The GFC had devastating micro- and macroeconomic impacts in developed and developing countries [14,15,[17], [18], [19],^49^]. The GFC significantly lags on various economic activities in different countries, including aggregate import demand [14,15,18,[50], [51], [52], [53]]. An impact from the global crisis (i.e., the GFC and pandemic) may prevail for at least ten years in an affected economy [21,22]. Various countries frequently changed their fiscal and monetary policies to control the effects of the GFC [14,15,18]. Consequently, the foreign exchange rate, domestic production, unemployment rate, inflation rate, and volume of external trade (imports and exports) in different countries changed over time [15,18]. Micro- and macroeconomic indicators of a small open economy like Bangladesh are likely more vulnerable to change amid the GFC [15,54]. The elasticities of determinants of import demand might also be changed in the affected countries. For example, a few studies revisited the elasticities of macroeconomic indicators, including the import demand in various countries due to the GFC [12,23]. For efficient import-related policy implications, countries must have post-GFC changed elasticities of the determinants, including disaggregate income components, of the import demand.

Only the aforementioned study in Bangladesh explored the elasticities of disaggregated income components of import demand using a sample of annual data from 1978 to 2008 [3]. Thus, the study could not capture any probable change in import demand elasticities due to the GFC. But the country needs post-GFC elasticities in import determinants for efficient import-related policy implications. However, a couple of studies on bilateral export and import demand in Bangladesh using annual data from 1981 to 2015 identified that trade openness significantly impacts import demand in response to changes in world prices [55,56]. Another study explored the elasticities of domestic credit to the private sector (financial development) and income (transformed to quadratic data using the quadratic match-sum method) to import demand in Bangladesh using the data from 1984 to 2014 and found a significant bidirectional impact between import and financial development in the short- and long-run [57]. Therefore, the findings of this study, in exploring the post-GFC short- and long-run elasticities of import determinants, including disaggregated income components, fill the knowledge gaps in the literature.

This study uses several econometric techniques, such as the EG procedure, VAR, and ARDL methods, for efficient results essential for relevant policy implications. Based on the variables’ characteristics (i.e., order of integration) and diagnostic test results, this study uses the best error correction mechanism (ECM) for efficient short-run elasticities of import determinants in the country.

### 2.1 Theoretical framework

This study estimates the long-run cointegrating relationship between import demand and its determinants in Bangladesh. The theoretical framework evolved based on the imperfect substitute demand model of the Marshallian total demand function for aggregate imports [26].

(1) $$M_{t}=f\;\left(Y_{t},RP_{t}\right),f_{1}\;>\;0,f_{2}\;<\;0$$

In Eq. (1), *M* = quantity of import demand, *Y* = real income, *RP* = relative prices (nominal import divided by nominal GDP), *t* = time, and *f*_*i*_ (where *i* = 1, 2) is the expected partial derivatives.

This study first shows the import demand as a traditional function of real income and relative prices, including foreign currency reserves. The ‘foreign currency reserve’ is an essential unconventional factor that indicates a visible foreign currency payment capacity for a developing country like Bangladesh [3,46]. Thus, the first model of the import demand function is specified as follows:

(2) $$M_{t}=f\;\left(Y_{t},RP_{t},R_{t}\right),f_{1}\;>\;0,f_{2}\;<\;0$$

In Eq. (2), *M* = quantity of import demand, *Y* = real income, *RP* = relative prices, *R* = foreign currency reserves, *t* = time, and *f*_*i*_ (where *i* = 1, 2) is the expected partial derivatives. Hence, the real income (*Y*) is (real) total GDP.

Following the existing literature and directions to avoid aggregation bias in the estimation [3,9,10,12,39], this study considers the disaggregate income components, such as the final private consumption and government expenditures, investment expenditures on goods, and exports, instead of GDP as unconventional determinants [3,9,10,12,35]. Thus, the functional form is as follows:

(3) $$M_{t}=f\;\left(FCG_{t},I_{t},X_{t},RP_{t},R_{t}\right),f_{1}\;>\;0,f_{2}\;<\;0$$

In Eq. (3), *M* = quantity of import demand, *FCG* = real final private consumption and government expenditures, *I* = real final investment expenditures on goods, *X* = real export demand, *RP* = relative prices, *R* = foreign currency reserves, *t* = time, and *f*_*i*_ (where *i* = 1, 2) is the expected partial derivatives.

## 3 Materials and methods

This study uses a sample of annual data from the fiscal years 1979–1980 (1980) to 2020–2021 (2021) because the quarterly data for all determinants of import demand are not available in Bangladesh. The total number of observations is 42. The sources of data are the ‘*World Development Indicators*’ of the World Bank [58], different issues of the ‘*Statistical Yearbook of Bangladesh*’ published by the Bangladesh Bureau of Statistics [24], and various issues of the ‘*Monthly Economic Trends*’ published by the Bangladesh Bank [59]. All variables are in real terms (the base year is 2010), and the data are in local currency.

This study does not use the variable of aid or grants as a determinant of import demand because the aid and grants come at a specific time or year to meet a specific purpose (e.g., natural disaster management). Thus, the trend of the aid variable is absent, or, in other words, the aid variable is stationary at its level. Thus, the results, including the aid variable, might be erroneous with other non-stationary variables [3]. Furthermore, this study excludes the variable ‘remittance’ as a factor of import demand because the remittance is indirectly included with the foreign currency reserves [3].

Bangladesh utilized the Trade Liberalization Policy from 1976 to 1982 [60]. The pace of trade liberalization accelerated during the 1980s after the country introduced the International Monetary Fund (IMF)-initiated structural adjustment programs [60]. Further, this trade liberalization gained momentum in the early 1990s by considerably reducing quantitative trade restrictions, tariff rates, and the gradual conversion of exchange rate policy (from a fixed to a managed exchange rate) [33,61]. So, there might be an impact of this trade liberalization on aggregate import demand in Bangladesh. It is noticeable that this study uses dummy TL = 0 before the year 1992 and TL = 1 from the year 1992 to show the impact of trade liberalization on aggregate import demand because the year 1992 is suggested by available literature as a drift for effective structural adjustment in Bangladesh [33,47].

It is customary to take the logarithmic form of all dependent and independent variables so that the elasticity of import demand becomes meaningful to explain. Thus, the empirical models in this study are as follows:

Model 1

(4) $$\ln\;M_{t}=\alpha_{0}+\alpha_{1}\;\ln\;Y_{t}+\alpha_{2}\;lnRP_{t}+\alpha_{3}\;\ln\;R_{t}+\alpha_{4}\;TL_{t}+\varepsilon_{t}$$

In Eq. (4), *M* = real import, constructed by dividing the total value of import by the import value index after necessary adjustment to the base year 2010, *Y* = constant GDP in the base year 2010, and *RP* = relative prices of imports (constructed by the nominal imports divided by the nominal GDP at the base year 2010), *R* = foreign currency reserves except gold, *TL* = trade liberalization dummy, *t* = time, *α1*, *α2*, *α3*, and *α4* are the coefficients of interest, and *ε* is an error term.

Model 2

(5) $$\ln\;M_{t}=\beta_{0}+\beta_{1}\;lnFCG_{t}+\beta_{2}\;\ln\;I_{t}+\beta_{3}\;\ln\;X_{t}+\beta_{4}\;lnRP_{t}+\beta_{5}\;TL_{t}+u_{t}$$

In Eq. (5), *M* = real import, *FCG* = real final private consumption and government expenditures, *I* = real final investment expenditures on goods, *X* = real export demand, *RP* = relative prices of imports, *TL* = trade liberalization dummy, *t* = time, *β*_1_, *β*_2_, *β*_3_, *β*_4_, and *β*_5_ are the coefficients of interest, and u is an error term.

In time series regression analysis, one of the critical assumptions is that the series is stationary. It is a fundamental rule to determine whether the time series contains the problem of a unit root. The static data are consistent with economic theory and can give a good estimation, but the non-stationary data give distorted and misleading results [62]. The study checks the stationarity of every series utilizing the Augmented Dickey-Fuller (ADF) and Phillips-Peron (PP) tests. Further, this study utilizes different estimation strategies, such as the EG [63], VAR [64], and ARDL [65] techniques, for a long-run cointegrating relationship between the import demand and its determinants in Bangladesh. Before using different econometric techniques, the study explains the variables’ notations, measurements, and sources in Table 1.

**Table 1:** Description of the used variables.

Variable	Notation	Measurement (in local currency)	Sources
Real aggregate import	M	Dividing the total value of import by the import value index after necessary adjustment to the base year 2010	World Development Indicators, Statistical Yearbook of Bangladesh, and Monthly Economic Trends
Real income or real Gross Domestic Product (GDP)	Y	Constant GDP in the base year 2010	
Relative import price	RP	The nominal import divided by the nominal GDP at the base year 2010	
Real final private consumption and government expenditures	FCG	Constant FCG in the base year 2010	
Real final investment expenditures on goods	I	Constant I in the base year 2010	
Real exports	X	Constant X in the base year 2010	
Foreign currency reserves	R	Total foreign currency reserves excluding gold	

### 3.1 Empirical results

#### 3.1.1 Unit root test

The study examines all series individually using the ADF and PP tests. The null hypothesis is that the series is non-stationary, and the critical values are at the 5% significance level. The results indicate that all series with an intercept (without trend) except real income (LY) are non-stationary at the level and stationary at the first difference through the ADF test. All series (without trend) are non-stationary at the level and stationary at the first difference through the PP test (Table 2). Thus, the results conclude that the series (without a trend) is stationary at first-difference I (1). Hence, the VAR technique is more appropriate. In addition, by applying both ADF and PP tests, the order of integration of the series with intercept and trend is mixed, such that LM, LX, and LI variables are stationary at level I (0) and LY, LRP, LR, and LFCG variables are stationary at first-difference I (1) (Table 2). Hence, the ARDL technique is more appropriate. The results of the ARDL technique are essential because this study focuses on the trend for a long-run cointegrating relationship, which may capture the impacts of the GFC.

**Table 2:** Unit root test and order of integration.

Variables	At level/first-difference	ADF Test		PP Test	
		With Intercept	With Intercept & Trend	With Intercept	With Intercept & Trend
LM	Level First difference	−2.32 −7.52**	−4.43** −4.63**	−1.95 −9.47**	−3.84** −14.96**
LY	Level First difference	6.18 −1.42	1.07 −4.49**	6.18 −3.52**	1.21 −9.51**
LRP	Level First difference	−0.01 −6.19**	3.17 −6.87**	−0.01 −6.19**	−3.26 −6.89**
LR	Level First difference	−1.93 −5.47**	−2.43 −5.38**	−1.93 −5.84**	−2.49 −5.62**
LX	Level First difference	−1.86 −9.77**	−5.09** −9.82**	−1.96 −14.87**	−5.04** −33.76**
LFCG	Level First difference	4.18 −3.62**	−0.77 −5.02**	4.36 −3.47**	−0.83 −5.02**
LI	Level First difference	−2.25 −12.34**	−2.01 −11.86**	−2.21 −15.62**	−6.34** −15.57**

Notes: ADF = Augmented Dickey-Fuller, and PP = Phillips-Perron. L denotes natural logarithm. The *, **, *** denotes significance at 10%, 5%, and 1% levels, respectively. Critical values for the ADF and PP tests are −2.63, −2.98, and −3.71 (with intercept), −3.23, −3.59, and −4.36 (with intercept and trend), which are taken as one-sided p values of MacKinnon (1996).

The ARDL technique's simplicity frees it from checking the order of integration of the variables, and the optimal lags are determined through a lag selection process [66]. The ‘F-bound test’ is used to check the existence of a long-run cointegration relationship between the series.

#### 3.1.2 Structural breakpoint

**Fig. 1:**
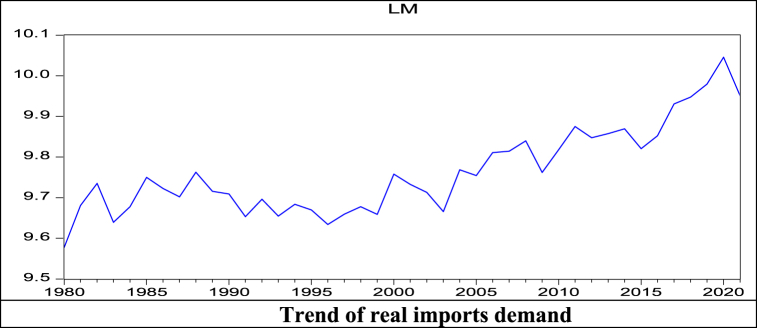
Trend of real imports demand.

The trend of real import has trough points in the years 1984, 2010, and 2021 due to political instability [33], the GFC [50], and the pandemic COVID-19 virus [67], which might be the structural breakpoints (Fig. 1). This study checks for an unknown breakpoint using Zivot-Andrew's breakpoint test. The results of the breakpoint test with either an intercept or an intercept and trend suggest no breakpoint in the trend of real import demand in Bangladesh. Studies using annual data from 1978 to 2008 also did not find a breakpoint in the country's import demand trend [3].

#### 3.1.3 Cointegrating long-run relationships

The study first utilizes the EG procedure on Models 1 and 2 to show the long-run relationship between import demand and its determinants. It checks for the cointegration between series using the ADF test on EG's residual at level and one-lag. The statistical values for Models 1 and 2 are lower than the critical values. Thus, the ADF tests on EG's residuals prove that every model has at least one cointegrating vector, establishing a long-run relationship between the import demand and its determinants in the country (panel B of Table 3).

**Table 3:** Cointegrating long-run estimations using the Engle-Granger (Fully Modified Least Square (FMOLS)) procedure.

Panel A: Variables	Model-1	Model-2
LY	1.03*** (0.08)	–
LRP	−0.49*** (0.09)	−0.28*** (0.01)
LR	−0.009** (0.004)	–
LX	–	0.40*** (0.12)
LFCG	–	0.16 (0.16)
LI	–	0.32*** (0.12)
Dummy(TL)	0.07*** (0.02)	0.06*** (0.02)
C	−3.30*** (1.04)	−0.77 (1.05)
**Panel B: Cointegration test through the ADF test on EG's residual**		
Residual at level	−5.84**	−5.40**
Residual at 1 lag	−4.73**	−5.41**
Diagnostic tests	R^2^ = 0.97 SER = 0.04 Normality = X^2^ 1.04 (prob. 0.59)	R^2^ = 0.98 SER = 0.03 Normality = X^2^ 0.33 (prob. 0.84)

Notes: Standard errors in parentheses, ***p < 0.01, **p < 0.05, and * p < 0.1. Critical values for the ADF unit root test at the 5% significance level are −4.35 for model-1 and -4.76 for model-2, which are taken from MacKinnon (1991).

This study uses a trace and max-eigenvalue-based VAR model to find a cointegrating long-run relationship for import demand. Hence, the study uses a 2-lag for Model 1 and a 3-lag for Model 2, based on the Akaike information criterion (AIC) and Schwarz information criterion (SC). Both trace and max-eigenvalues show that model 1 has one cointegrating vector and model 2 has two cointegrating vectors. These vectors establish the long-run relationships between import demand and its determinants (panel B of Table 4).

**Table 4:** Cointegrating long-run estimations by using the Vector Autoregression (VAR) model.

Panel A: Variables		Model 1			Model 2		
LY		1.03 (0.05)			–		
LRP		−0.22 (0.05)			−0.87 (0.12)		
LR		−0.008 (0.002)			–		
LX		–			0.39 (0.22)		
LFCG		–			0.77 (0.17)		
LI		–			0.75 (0.14)		
C		−3.21			−6.79		
**Panel B: Cointegration test**							
**Null**	**Alternative**	**Eigenvalue**	**Trace Statistics**	**Max-Eigen** **Statistics**	**Eigenvalue**	**Trace Statistics**	**Max-Eigen** **Statistics**
r = 0 r ≤ 1 r ≤ 2 r ≤ 3 r ≤ 4	r = 1 r = 2 r = 3 r = 4 r = 5	0.65 0.34 0.31 0.07	68.58* (55.25) 31.07 (35.01) 15.98 (18.40) 2.72 (3.84)	37.51* (30.82) 15.09 (24.25) 13.27 (17.15) 2.72 (3.84)	0.81 0.78 0.49 0.12 0.05	142.91* (79.34) 83.94* (55.25) 30.34 (35.01) 6.63 (18.40) 1.95 (3.48)	58.96* (37.16) 53.60* (30.82) 23.71 (24.25) 4.67 (17.15) 1.95 (3.84)
Diagnostic tests		Observations = 39 (adjusted) R^2^ = 0.63 SEE = 0.03 Portmanteau = X^2^ 64.50 (p. 0.0001) Normality = X^2^ 3.23 (p. 0.91) LM = F 0.52 (p. 0.91) Hetero. = X^2^ 255.07 (p. 0.39)			Observations = 39 (adjusted) R^2^ = 0.98 SEE = 0.03 Portmanteau = X^2^ 179.70 (p. 0.000) Normality = X^2^ 2.57 (p. 0.98) LM = F 1.76 (p. 0.054) Hetero. = X^2^ 468.43 (p. 0.45)		

Notes: In panel A: standard errors in parentheses, ***p < 0.01, **p < 0.05, *p < 0.1. In panel B: r denotes the number of cointegrating vectors. Critical values are provided in the parentheses, which are taken from MacKinnon, Haug and Michelis (1999).

The study finally employs the ARDL technique on both models and checks for a cointegrating relationship by introducing the F-bound test. The test results indicate a long-run relationship between import demand and its determinants in every model (panel B of Table 5).

**Table 5:** Cointegrating long-run estimations using the Autoregressive Distributive Lag (ARDL) model.

Panel A: Variables	Model 1 (1, 0, 3, 1)	Model 2 (2, 2, 1, 1, 3)
LY	0.86*** (0.09)	–
LRP	−0.26** (0.10)	0.34* (0.17)
LR	−0.015*** (0.004)	–
LX	–	1.01*** (0.23)
LFCG	–	−0.96*** (0.29)
LI	–	−0.28 (0.22)
Trend	0.02*** (0.01)	0.02*** (0.01)
C	−1.10 (1.10)	14.70 (3.72)
**Panel B: Cointegration test through the F-Bound test**		
Statistics values	9.36	10.54
Critical values	I(0) I(1) 5%: 3.16 4.20 1%: 4.43 5.82	I(0) I(1) 5%: 4.04 4.51 1%: 5.60 7.17
Diagnostic tests	Observations = 39 R^2^ = 0.98 DW = 2.08 SER = 0.02 Normality = X^2^ 0.83 (p. 0.66) LM = 0.29 (p. 0.86) Hetero. = 12.40 (p. 0.25) ARCH = 0.09 (p. 0.95) Ramsey-reset = 0.30 (p. 0.58)	Observations = 38 R^2^ = 0.99 DW = 1.94 SER = 0.01 Normality = X^2^ 0.49 (p. 0.78) LM = 3.42 (p. 0.33) Hetero. = 13.99 (p. 0.52) ARCH = 5.52 (p. 0.14) Ramsey-reset = 0.40 (p. 0.53)

Notes: Models run with automated lag selection process in the statistical software (Eviews 10), P = probability.

Diagnostic tests suggest that both models using the EG, VAR, and ARDL approaches do not suffer from autocorrelation, serial correlation, or heteroscedasticity problems. The normality test suggests that the residuals of both models contain all the properties of a classical linear regression model. The Ramsey-reset test suggests that the models’ linear specifications are not incorrect (panel B of Table 3, Table 4, Table 5).

In Model 1 (with aggregate income), the income elasticity of import demand is 1.03 using the EG and VAR approaches and 0.86 using the ARDL approach. The price inelasticity of import demand is −0.49, −0.22, and −0.26 by applying the EG, VAR, and ARDL approaches, respectively. These relations among the variables of import demand in Bangladesh conform to economic theories. The foreign currency reserves-elasticity of import demand is negligibly negative at about −0.01 using all techniques (panel A of Table 3, Table 4, Table 5). This negative elasticity of foreign reserves is controversial in conventional wisdom. Because the availability of foreign exchange positively induces more imports [3,47], the unit coefficient of income restriction and weak exogeneity test indicate that the foreign currency reserves coefficient is positive (about 0.01) in the VAR and EG procedures. The chi-square's p-values in the VAR approach and the Wald test's F-statistics in the EG procedure are 0.721 and 0.723, respectively. Thus, the overall outcomes using all econometric techniques suggest that the import demand in Bangladesh is positively elastic (0.86–1.03) with income and is negative and moderately inelastic (0.22–0.49) with relative prices. The influence of foreign currency reserves is negligible in the long run. These relations between import demand and its conventional determinants are consistent with the previous findings in the country [3,30].

In Model 2 (with disaggregated income components), the export elasticity of import demand is 0.40, 0.39, and 1.01 using the EG, VAR, and ARDL approaches, respectively. Those elasticities of final private consumption and government expenditures, final investment, and relative prices are 0.16, 0.77, and −0.96; 0.32, 0.75, and −0.28; and −0.28, −0.87, and 0.34, respectively (panel A of Table 3, Table 4, Table 5). However, signs of LFCG, LI, and LRP in the ARDL technique do not conform to economic theories because a developing country's final private consumption, government expenditures, and final investment goods cannot negatively correlate with the import demand. The relative prices in the ARDL model cannot be positively associated with the import demand, and the export elasticity cannot be greater than 1. So, the ARDL technique for unconventional determinants in Model 2 needs to be more consistent. On the other hand, the results using the EG and VAR procedures conform to the economic theories and are consistent with the previous findings in the country [3]. The overall results indicate that the import demand of Bangladesh is positive and moderately inelastic with exports at 0.40, is positive and inelastic with private consumption and government expenditures at 0.16–0.77, and is positive and inelastic with final investment at 0.32–0.75 in the long run.

In addition, both models under the EG procedure indicate that trade liberalization has a little positive impact (0.06–0.07) on import demand in Bangladesh in the long run (panel A of Table 3). Both models using the ARDL technique also suggest that the trend has a negligibly positive (0.02) influence on import demand in the long run (panel A of Table 5).

#### 3.1.4 Weak-exogeneity test and check of the unit coefficient of income

The study utilizes the weak exogeneity test of Models 1 and 2 by applying the Johansen technique [68]; the results ensure a long-run cointegrating relationship in each model. The import demand is identified as a dependent variable, and all other determinants are identified as explanatory variables. The chi-square-based test result of weak exogeneity for Model 1 is 2.44 [prob. 0.295] and for Model 2 is 5.47 [prob. 0.102].

Furthermore, the study checks the ‘unit coefficient of income (*β*_1_ = 1)' restriction on model 1 and gets the chi-square test result of 0.19 [prob. 0.568], and the coefficients of income, relative prices, and foreign currency reserves are 1, -0.20, and 0.01, respectively. The study also checks for the unit coefficient of income restriction on model 1 using the Wald test in the EG procedure and finds the chi-square test result is 0.13 [prob. 0.721], and the F-statistic result is 0.13 [prob. 0.723]. These results of restrictions on Model 1 cannot reject the null hypothesis of the unit coefficient of income of import demand in Bangladesh, which is consistent with the previous result in the country [3].

#### 3.1.5 ECM and Robustness check

The study explores the best short-run dynamic equation of import demand by applying all econometric techniques to both models. This study uses the variables' first-difference form, following the estimation procedure of Hendry's ‘general to specific’ modeling approach [69]. The general equations of models 1 and 2 are as follows:

Model 1:

(6) $$\Delta lnM_{t}=\alpha_{0}+\sum_{i=1}^{2}\alpha_{i}\Delta \ln M_{t-i}+\sum_{i=0}^{2}\beta_{i}\Delta \ln Y_{t-i}+\sum_{i=0}^{2}\rho \Delta \ln{\left(RP\right)}_{t-i}+\sum_{i=0}^{2}{ᴓ}_{i}\Delta \ln\;R_{t}+\gamma ECM_{t-1}+\sigma \;Dummy\left(TL\right)+\lambda Dummies+\varepsilon_{1}$$

(7) $$Model\;2:\Delta lnM_{t}=\alpha_{0}+\sum_{i=1}^{3}a_{i}\Delta \ln M_{t-i}+\sum_{i=0}^{3}b_{i}\Delta \ln\;X_{t-i}+\sum_{i=0}^{3}c\Delta \ln{\left(RP\right)}_{t-i}+\sum_{i=0}^{3}d_{i}\Delta \ln\;CG_{t}+\sum_{i=0}^{3}e_{i}\Delta \ln\;I_{t}+gECM_{t-1}+hDummy\left(TL\right)+wDummies+u_{1}$$

This study sequentially eliminates all non-significant lags and variables from the general equation. The results from different econometric techniques are reported in Table 6, Table 7 for models 1 and 2, respectively.

**Table 6:** The Equilibrium Correction Mechanism (ECM) on Model-1 (conventional determinants).

Variables	Engle-Granger	VAR	ARDL
DLY	1.63*** (0.38)	0.99* (0.50)	1.18** (0.50)
DLRP	−0.58*** (0.06)	−0.49*** (0.09)	−0.47*** (0.09)
DLRP(-1)	0.20*** (0.07)	0.38*** (0.10)	0.50*** (0.09)
ECM(-1)	−0.70*** (0.10)	−0.52*** (0.16)	−0.79*** (0.24)
Dummy(TL)	−0.008 (0.011)	−0.006 (0.01)	−0.01 (0.01)
Long-run aggregate demand for imports	LM = 1.03 LY – 0.49 LRP – 0.009 LR	LM = 1.03 LY – 0.22 LRP – 0.008 LR	LM = 0.86 LY – 0.26 LRP – 0.015 LR
Diagnostic tests	Observation: 40 R-squared: 0.86 DW: 1.89 AIC: 4.83 SC: 4.57 Normality: 1.69 (p. 0.43) LM: 1.07 (p. 0.58) Hetero.: 2.78 (p. 0.83) ARCH: 3.73 (p. 0.15) White: 21.12 (p. 0.27) Ramsey-reset: 2.23 (p. 0.14)	Observation: 38 R-squared: 0.76 DW: 1.44 AIC: 4.37 SC: 4.10 Normality: 0.23 (p. 0.88) LM: 6.77 (p. 0.03)** Hetero.: 7.00 (p. 0.32) ARCH: 0.38 (p. 0.82) White: 16.14 (p. 0.51) Ramsey-reset: 2.34 (p. 0.13)	Observation: 38 R-squared: 0.74 DW: 1.48 AIC: 4.32 SC: 4.05 Normality: 0.43 (p. 0.80) LM: 6.19 (p. 0.04)** Hetero.: 3.75 (p. 0.70) ARCH: 0.66 (p. 0.71) White: 9.66 (p. 0.88) Ramsey-reset: 5.55 (p. 0.02)**

Notes: Standard errors in parentheses; ***p < 0.01, **p < 0.05, *p < 0.1; TL = Trade Liberalization; OL = Outlier (the EG used year 2004 and 2017, and the ARDL used year 2004 as outliers).

**Table 7:** The ECM on Model-2: unconventional determinants.

Variables	Engle-Granger	VAR	ARDL
DLM(-1)	**-**	**-**	0.48*** (0.10)
DLM(-2)	–	−0.37*** (0.13)	–
DLX	0.54*** (0.06)	0.61*** (0.09)	0.68*** (0.07)
DLX(-1)	–	0.24*** (0.07)	–
DLX(-2)	–	0.33*** (0.09)	–
DLRP	−0.63*** (0.07)	−0.25** (0.09)	−0.46*** (0.08)
DLRP(-1)	0.21*** (0.06)	0.47*** (0.09)	0.65*** (0.09)
DLRP(-2)	0.19*** (0.06)	–	–
DLFCG	1.35*** (0.31)	1.21*** (0.42)	1.90*** (0.32)
DLFCG(-1)	−0.80** (0.33)	−1.59*** (0.50)	−1.77*** (0.39)
DLI	−0.31** (0.12)	–	−0.57*** (o.13)
DLI(-2)	0.35*** (0.09)	–	–
DLI(-3)	0.09** (0.04)	–	0.33*** (0.09)
ECM(-1)	−0.66*** (0.13)	−0.06 (0.17)	−1.00*** (0.26)
Dummy(TL)	−0.0003 (0.007)	−0.007 (0.01)	0.0005 (0.007)
Dummy(OL)	−0.05*** (0.01)	–	−0.02 (0.01)
Long-run aggregate demand for imports	LM = 0.40 LX – 0.28 LRP + 0.16 LFCG + 0.32 LI	LM = - 0.39 LX – 0.87 LRP + 0.77 LFCG + 0.75 LI	LM = 1.01 LX + 0.34 LRP - 0.96 LFCG - 0.28 LI
Diagnostic tests	Observations: 38 R-squared: 0.94 DW: 2.27 AIC: 5.51 SC: 4.98 Normality: 1.96 (p. 0.37) LM: 3.23 (p. 0.35) Hetero.: 12.97 (p. 0.37) ARCH: 1.21 (p. 0.74) Ramsey-reset: 0.14 (p. 0.70)	Observations: 37 R-squared: 0.85 DW: 1.83 AIC: 4.79 SC: 4.34 Normality: 2.72 (p. 0.25) LM: 2.22 (p. 0.52) Hetero.: 5.15 (p. 0.88) ARCH: 2.23 (p. 0.52) Ramsey-reset: 0.69 (p. 0.41)	Observations: 38 R-squared: 0.93 DW: 2.14 AIC: 5.32 SC: 4.83 Normality: 1.09 (p. 0.57) LM: 0.87 (p. 0.83) Hetero.: 7.36 (p. 0.76) ARCH: 1.42 (p. 0.69) Ramsey-reset: 0.15 (p. 0.69)

Notes: Standard errors in parentheses, ***p < 0.01, **p < 0.05, *p < 0.1. OL = outlier (the EG used year 2009 and 2017, and ARDL used year 2009 as outliers).

In Model 1, the VAR and ARDL approaches’ ECMs suffer from the autocorrelation problem. The Ramsey-reset test of the ARDL technique indicates that the linear function is not well specified (Table 6). However, the results of VAR and ARDL are still unbiased and consistent but inefficient [70]. By using the ECM of the EG procedure, it is possible to derive the unbiased, consistent, and effective parsimonious equation of Model 1 as follows:

(8) $$\Delta \ln\;M_{t}=1.63\;\Delta \ln\;Y_{t}-0.58\;\Delta lnRP_{t}+0.20\;\Delta lnRP_{t-1}-0.70\;ECM_{t-1}$$

In Model 2, the ECM of the VAR model is not statistically significant. The ECM of ARDL indicates that the import demand of Bangladesh is always in the equilibrium position, which is not consistent because the import demand of a developing country with a managed exchange rate [61] can sometimes be in the equilibrium position (Table 7). As a result, using the ECM of the EG procedure, it is also possible to derive the satisfactory parsimonious equation of Model 2:

(9) $$\Delta lnM_{t}=0.54\;\Delta lnX_{t}-0.63\;\Delta lnRP_{t}+0.21\;\Delta lnRP_{t-1}+0.19\;\Delta lnRP_{t}-2+1.35\;\Delta lnFCG_{t}-0.80\;\Delta lnFCG_{t-1}-0.31\;\Delta lnI_{t}+0.35\;\Delta lnl_{t-2}+0.09\;\Delta \ln l_{t-3}-0.66\;ECM_{t-1}$$

The parsimonious (8), (9) indicate that relative price elasticity (RP) is a significant determinant of import demand in Bangladesh in the short run. Hence, the price elasticity of import demand is roughly −0.60. Eq. (8) suggests that the income elasticity of import demand is higher at 1.63 in the short run. Eq. (9) suggests that the export elasticity of import demand is 0.54, and the (net) final private consumption and government expenditures elasticity is 0.55 in the country. However, the short-run final investment elasticity of import demand appears negative in the current year. The investment elasticity of import demand appears positive with a 2- and 3-year lag. Thus, the net effect of final investment on import demand is also positive, at 0.11 in the short run.

The ECM is negative and statistically significant, which is necessary for the models’ stability. The adjustment speed of back-to-equilibrium is minus 0.66–0.70, which implies a very rapid adjustment from short-run disequilibrium to long-run equilibrium. This rapid adjustment is consistent with the studies in Bangladesh and India [3,10].

Diagnostic test results suggest that both models (1 and 2) are stable using the EG procedure. Where autocorrelation, serial correlation, or heteroscedasticity problems are absent. The normality test suggests that the residuals of both models contain all the properties of classical linear regression. The Ramsey-reset test suggests that the models’ linear specifications are not incorrect (Table 6, Table 7).

This study also graphically examines models’ structural stability using the cusum, cusum of the square, and beta coefficients. The graphical representations suggest that the residuals of Models 1 and 2 are within ±2 standard errors, the beta coefficients of Models 1 and 2 are also within ±2 standard errors, and the coefficient movements are minimal (Appendix: Figures A1–A4). Thus, the overall test results suggest that both models are stable and appropriate for policy implications.

## 4 Discussion

The composition of imported volumes should be fine in the early developing stages of an economy because most of the imported goods are required for consumption, production, or both [71]. Bangladesh has been experiencing a trade deficit since its independence in 1971 [24,55]. The first two decades were due to the excess import of necessary goods (i.e., foods and consumer goods), raw materials, and machinery for exporting items, especially ready-made garments [55]. Thus, as a small open economy, Bangladesh requires more imported capital goods for its exporting firms to meet the expanded export demand. Although price is a concerning issue in economic analysis, price and income are essential factors in the case of import demand [33]. The income elasticity was always higher than the relative prices of imported goods and services in Bangladesh [3,[46], [47], [48]]. The elasticities of some unconventional determinants of import demand, i.e., foreign currency reserves, aggregate exports, and investment, were significant in the long run [46,48], but only relative prices and exports were significant in the short run [3]. These elasticities of import demand's determinates were demonstrated based on the data up to 2008 and could not capture any probable impact of the GFC.

### 4.1 Conventional determinants of import demand

This study finds a long-run cointegrating relationship between import demand and its traditional determinants (income and relative prices) through all three econometric techniques (i.e., EG, VAR, and ARDL). Without the trend, both EG and VAR techniques demonstrate unbiased and consistent results with the expected signs of the determinants. The Wald test and the unit coefficient of income restriction confirm the study's discovery of a positive unit income elasticity using the EG and VAR techniques. The relative price elasticity is negative and inelastic, i.e., the EG procedure shows −0.49, and the VAR technique shows −0.22. The positive unit coefficient of income and negative inelasticity of relative prices are consistent with the available studies in Bangladesh [3].

This study considers a sample of annual observations up to 2021, including the trend term, so that the estimated results can capture the probable impacts of the GFC. The order of integration of the determinants, including intercept and trend, is mixed. The VAR technique shows that one cointegrating vector exists in the long run. Thus, the ARDL technique is more appropriate for the conventional import demand function with a trend. In the long run, the ARDL technique shows that the income and relative price elasticities are 0.86 and −0.26, respectively. The trend is positive but negligible at 0.02. These inelastic (positive) income and (negative) relative price elasticities are consistent with the findings (0.93 and −0.29, respectively) in a study in Bangladesh [30].

In a short-run dynamic analysis, this study finds that import demand is positively responsive (0.99–1.62) to income and negatively responsive (0.47–0.58) to relative prices. However, the EG procedure's error term is white noise, while other error terms (VAR and ARDL) are suboptimal or inefficient due to autocorrelation problems [70]. The EG procedure's unbiased, consistent, and efficient findings suggest that import demand's income and price elasticities are 1.63 and −0.58, respectively. The ECM is statistically significant, with an expected minus sign at −0.70, indicating a rapid adjustment from a short-run disequilibrium toward a long-run equilibrium. The short-run price inelasticity and ECM are close to the findings (−0.70 and −0.66, respectively) in available studies in Bangladesh [3].

### 4.2 Unconventional determinants of import demand

This study finds that the VAR and EG procedures demonstrate unbiased and consistent results, but most of the results using the ARDL technique need to be consistent with economic theory. Hence, the VAR technique is more appropriate since two cointegrating vectors exist in the long run. The elasticities of relative prices, exports, final private consumption and government expenditures, and investment expenditures on goods are −0.87, 0.40, 0.77, and 0.75, respectively. The coefficients of exports and government expenditures are close to the findings (0.30 and 0.62, respectively) in a study in Bangladesh [3].

The short-run dynamic analysis suggests that the results, including unconventional determinants using the EG procedure, are unbiased, consistent, and efficient (the reasons for the inconsistency of other ECMs are discussed earlier). The short-run elasticities of relative price, export, final private consumption and government expenditure, and investment expenditures on goods are −0.54, 0.63, 1.35, and 0.11, respectively. The ECM is also significant, with a negative sign at −0.66, which implies a rapid adjustment process from a short-run disequilibrium to a long-run equilibrium. The coefficients of relative price and ECM are very close to the findings (−0.66 and −0.69, respectively) in a study in Bangladesh [3]. However, the short-run export elasticity is almost triple the previous finding for the country. The long-run elasticities of disaggregated income in this study are also close to the findings in Ghana [9].

### 4.3 The trend of income elasticity

With the sample before 2000, the long-run income elasticity was higher than unity at 1.6–2.0 [[46], [47], [48]]. Later, with a sample of annual data up to 2008, all studies [3,30] except one [33] found a reduced long-run income elasticity to the unit coefficient [3] and less than the unit coefficient at 0.93 [30]. However, one study found a higher than a unit coefficient of income of 1.7–1.9 [33]. This study finds a unit coefficient of income at the intercept and less than a unit coefficient of income (0.86) with an intercept and a trend in the long run. Thus, the income elasticity reduces to less than a unit considering the impact of the GFC over time, which is consistent with the finding in Türkiye [12]. The main arguments behind the higher income elasticity before 2000 are that people were highly responsive to importing luxurious goods [48]. The economy depended highly on essential goods [47,48] and raw materials for the exporting firms [3,33,48]. The income elasticity of import demand decreased during the last two decades while the country could produce more necessary goods and substitute some raw materials for the exporting sector [3,30].

**Fig. 2:**
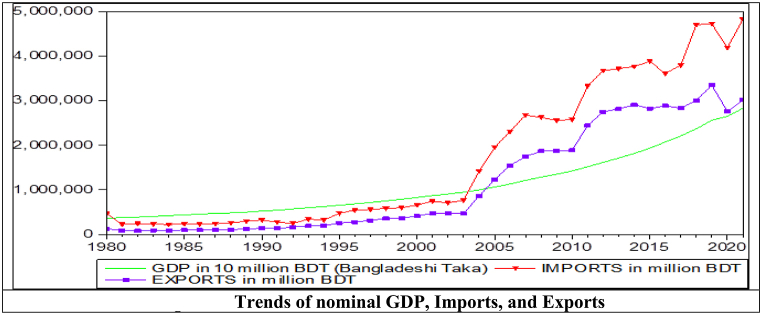
Trends of nominal GDP, Imports, and Exports.

The GFC could reduce import demand in 2010. However, after that time, the impact was released on the country (Fig. 2) for several micro- and macroeconomic initiatives, i.e., higher domestic production, food security, expansion of exports, and higher remittance earnings [72]. As a result, the income elasticity is still close to the unit coefficient in the long run. Additionally, this study finds that the short-run income elasticity of import demand is greater than unity at 1.63. The probable reasons for higher short-run income elasticity are that exporting firms import many capital goods to meet global demand, and people also import luxurious goods to meet domestic demand.

### 4.4 The trend of relative price elasticity

The long-run negative price inelasticity was in the range of 0.40–0.52 [[47], [48], [49]] and 0.29–0.58 [3,30] with the samples before 2000 and 2009, respectively. However, most studies found negative price inelasticity around 0.50 in the long run [3,48]. This study also finds negative price inelasticities between 0.26 and 0.87. The range of negative price inelasticity widens due to the increased substitution rate of importable goods and services in the economy. The same implication also applies in the short run, where this study finds a negative price inelasticity in the range of 0.54–0.58, slightly higher at 0.66–0.70 before 2009 [3].

### 4.5 The trend of unconventional determinants’ elasticities

This study finds an increased export elasticity of import demand from 0.30 to 0.40 in the long run. It is a good sign for Bangladesh because exports increased almost 3.9 times from 2008 to 2021 (Fig. 2). However, the increase in export elasticity was only 0.10, suggesting that domestic products are most likely a significant substitute for the raw materials used to make exportable goods. The export-inelasticity of import demand indicates that about 40% of total exports depend on imported capital goods and raw materials. The long-run elasticities of private consumption and government expenditures (FCG) and expenditure on investment goods (I) are 0.62 and 0.05, respectively, with a sample up to 2008 [3]. This study finds that those elasticities increased to 0.77 and 0.75, respectively. The probable reasons for high elasticities are that the government and the general public demand more imported luxurious goods for consumption and raw materials for exporting firms. Large- and medium-scale investment projects import considerable goods and services into the economy, so the gap between imports and exports has widened annually (Fig. 2). This study finds the short-run export elasticity at 0.63, which was 0.23 with a sample up to 2008 [3].

This study includes foreign exchange reserves as an essential unconventional determinant of import demand in Bangladesh. The long-run coefficient of exchange reserves in all econometric techniques is very low, in the range of 0.008–0.015. The sign is unexpectedly negative, similar to the country's previous studies [3,47]. However, the sign of exchange reserves appears positive (0.01) in the case of the exogeneity check and the unit coefficient of income test. A study also demonstrated a positive sign of reserves with the same processes in the country [3]. Thus, this study concludes that foreign exchange reserves have a negligible positive influence on import demand in the economy.

### 4.6 Impact of trade liberalization

The long-run impact of trade liberalization on import demand using samples up to 1994 and 2008 was positive at 0.10 and 0.05, respectively [3,47]. This study also finds the impact of trade liberalization at 0.07, which is somewhere between the previous results. These results suggest that trade liberalization has historically had a lower positive impact on import demand. However, the impact of trade liberalization is higher on imports than exports [33,73]. The main reason for higher import demand is the import of raw materials and capital goods for exporting firms [33].

### 4.7 Limitations

This study has some limitations that should be considered concerning its policy implications. For instance, this study cannot address temporal bias in the estimation since it analyzes a sample of annual data, which cannot capture any short-term seasonal variations. Quarterly data are better for minimizing temporal bias, but quarterly data are unavailable in Bangladesh. Another limitation of this study is aggregation bias (although this study minimizes aggregation bias in the determinants of import demand by introducing income components) because this study uses aggregated import demand as the outcome variable. This study suggests more studies on disaggregated import demand (e.g., item-wise imported goods and bilateral import demand), which can minimize the aggregation bias in the outcome variable. There might be different relationships between import demand and its determinants in some giant economies where Bangladesh generally meets a significant portion of its total imports, e.g., China and India. For instance, despite a massive reduction in import demand due to the GFC, the trend of imports from BRICS countries (Brazil, Russia, India, China, and South Africa), especially China, increased steadily [53]. Therefore, bilateral import demand models with major trading partners are also crucial for effective import-related policies in the country.

## 5 Policy implications

The production capacity of Bangladesh indicates that the exchange rate plays a vital role in determining the country's import demand because most of the exporting firms are importing capital goods for their expected supply of exports [3,33,73]. In addition, due to the reduction of quantitative restrictions, i.e., tariffs and quotas [3,33], Bangladesh has been pursuing an export-led trade policy for its economic growth strategy. It has maintained an active exchange rate policy since the 1980s [61]. This active exchange rate policy's primary goals are maintaining Bangladeshi products' competitiveness in the global market and maintaining a viable position in the country's external account [3,74,75]. The depreciation of domestic currency can immediately increase the demand for domestic goods in foreign markets; as a result, exporting firms will also demand importing capital goods to meet global demand [3]. Although Bangladesh could manage the impact of the GFC by implementing some needed economic policies [72], the ongoing pandemic and war (between Russia and Ukraine) have put extra pressure on stagflation in the global economy, including Bangladesh [25]. Thus, the policy of domestic currency depreciation to boost trade is a matter for future study because the ongoing war has already negatively impacted the fuel market [76], remittance earnings, exports, import demand, and foreign currency reserves [25]. Moreover, the ongoing pandemic has increased poverty, inequality, inflation, and unemployment in the country [25,67,77,78].

However, this study offers some policies based on the findings and in consideration of the limitations mentioned earlier. This model would be helpful in forecasting import demand for the country since income elasticity and relative price inelasticity have been steady for a long time [33].

Bangladesh's exports' global income elasticity is higher than the import demand's income elasticity [33,73]. The findings, including trend analysis of import demand determinants, indicate that import demand's export elasticity has slightly increased to 0.40. Trade liberalization and foreign currency reserves have little positive influence on aggregate import demand. So, Bangladesh, as a small open economy, can require more imported capital goods for its exporting firms to meet the expanded export demand.

The trend of less-than-unit income elasticity and the rising remittance earnings, exports, and foreign currency reserves suggest that the trade deficit emerges slowly (Appendix: Figure A5). The non-zero price inelasticity of import demand indicates that domestic products substitute for the economy's importable goods. The imported items are not merely essential consumption goods but also capital and luxurious goods [33]. Thus, Bangladesh should take more trade liberalization initiatives, such as qualitative and quantitative tariffs, quotas, and VAT reductions, to increase export volumes in the economy. For instance, higher trade liberalization would facilitate exporting firms' ability to import more capital goods [1,79] so export volumes could be adequately increased.

However, the government and the general public should be concerned regarding the probable impacts of the ongoing pandemic and war on the economy. This study suggests further research on aggregate and disaggregate import demand, including the impact of the pandemic and war. At least in the short run, unproductive and luxurious importable goods and services for government expenditures, private consumption, and investment expenditures should be reduced as a precautionary measure. So extra unfavorable pressure from the ongoing pandemic and war on the foreign currency reserves and balance of payments can be mitigated.

## 6 Conclusion

Using a sample of annual data from 1980 to 2021, this study demonstrates the elasticities of import demand's determinants, considering the impacts of the GFC. The study analyzes the import demand of Bangladesh in the long run by utilizing different time series econometric techniques, such as the EG, VAR, and ARDL techniques. The study also explores the short-run parsimonious dynamic equation by adopting the best ECM of the EG procedure. It suggests that the speed of adjustment back to equilibrium is very rapid and requires less than five months.

The overall findings, including diagnostic tests' statistics and models' stability test results, suggest that Model 1 with all procedures (EG, VAR, and ARDL) and Model 2 with the EG and VAR procedures are stable in the long run. Income and relative prices are the significant determinants of import demand in Bangladesh's short and long runs. Considering the trend term in the estimations to capture the impacts of the GFC, this study finds that the income elasticity reduces over time. The components of income, namely, real exports, private consumption and government expenditures, and investment in goods, are inelastic (less than a unit coefficient) with an expected positive sign in the long run. The export-inelasticity of import demand indicates that exports heavily depend on imported raw materials and capital goods. All conventional and unconventional determinants of import demand are also statistically significant, with expected signs in the short run.

The non-zero (negative) price inelasticity of import demand indicates that the competitiveness of domestic products substitutes for the importable goods in the economy. In addition, foreign currency reserves and trade liberalization have a slightly positive influence on import demand in the long run. The relevant policymakers in Bangladesh can use these stable models, which account for the impact of the GFC on import-related policy implications.

## Funding

The author did not receive any funding for this study.

## Availability of data and materials

Data are available at “World Development Indicators” of the World Bank (https://databank.worldbank.org/source/world-development-indicators), different issues of the “Statistical Yearbook of Bangladesh” published by the Bangladesh Bureau of Statistics (https://www.bbs.gov.bd/site/page/29855dc1-f2b4-4dc0-9073-f692361112da/Statistical-Yearbook) and various issues of the “Monthly Economic Trends” published by the Bangladesh Bank (https://www.bb.org.bd/en/index.php/publication/publictn/3/10).

## Declaration of competing interest

The authors declare that they have no known competing financial interests or personal relationships that could have appeared to influence the work reported in this paper.
