# Supplementary material for: Aggregated imports and expenditure components in Bangladesh: A cointegration and equilibrium correction analysis
Source: Heliyon. 2023 Jun 20;9(6):e17417. doi: 10.1016/j.heliyon.2023.e17417 (PMC10361366; doi:10.1016/j.heliyon.2023.e17417)
Supplement: Multimedia component 1 [file mmc1.docx]

| 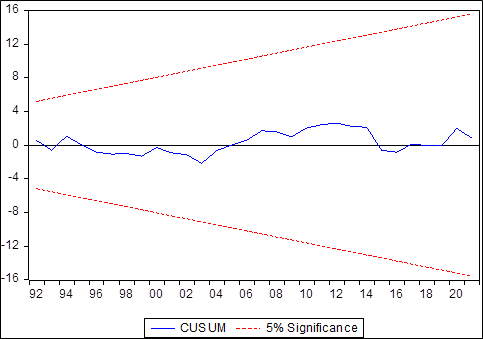 |  | 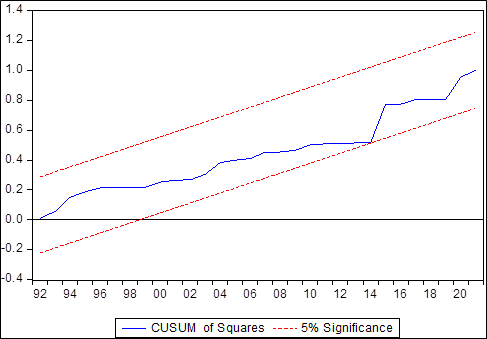 |
| --- | --- | --- |
| **Figure A1: Structural instability test on model-1** | | |

**Appendix:**

| 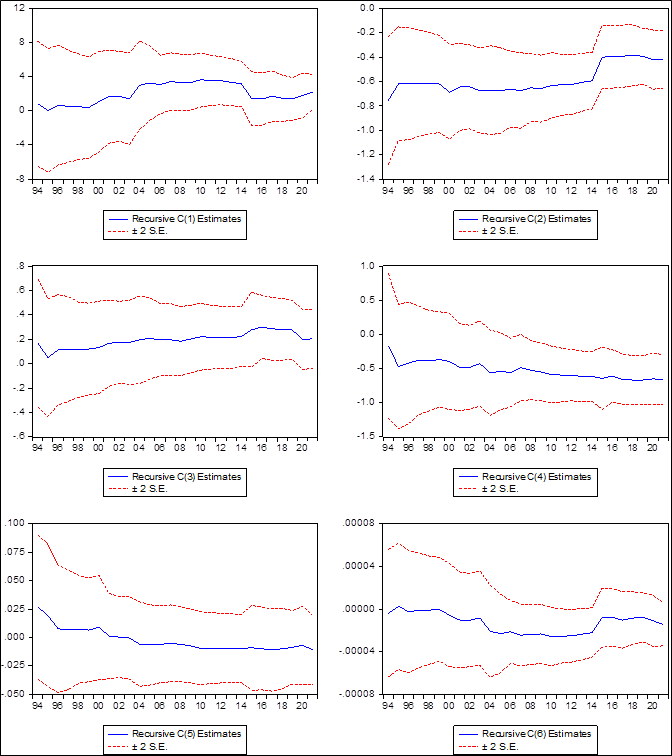 |
| --- |
| **Figure A2: Stability of beta coefficients of Model-1** |

| 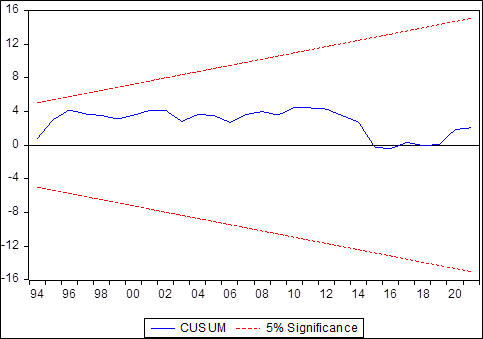 |  | 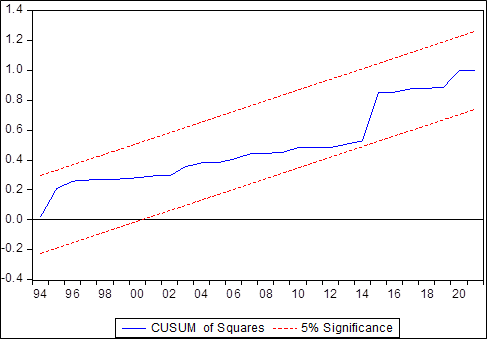 |
| --- | --- | --- |
| **Figure A3: Structural instability test on model-2** | | |

| 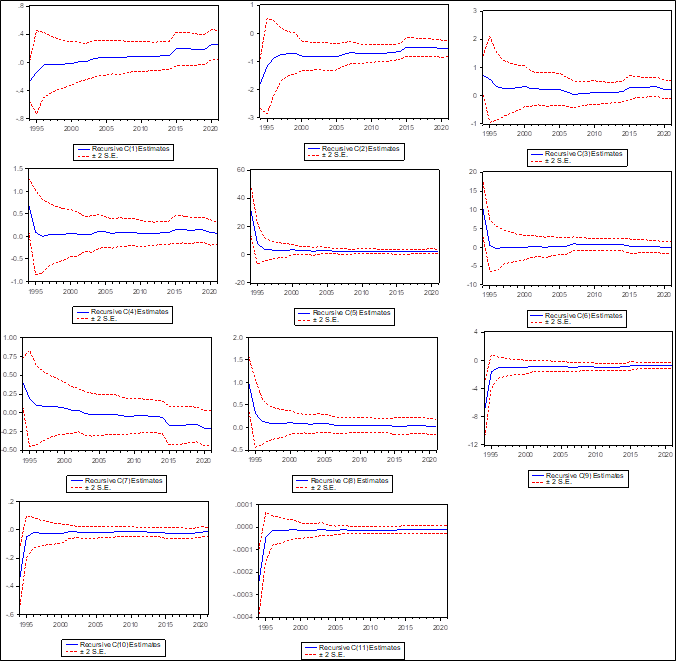 |
| --- |
| **Figure A4: Stability of beta coefficients of Model-2** |

|  |
| --- |
| Source: the Annual Report of Bangladesh Bank (BB, 2022) |
